# Automated Segmentation of Diffuse and Multifocal Nerve Enlargement in Immune-Mediated Neuropathy Using Temporal Deep Learning on Continuous Ultrasound Scans

**DOI:** 10.3390/diagnostics16121934

**Published:** 2026-06-22

**Authors:** Miho Akaza, Ryo Maeda, Tai Otani, Hirokazu Natsui, Tadashi Kanouchi, Yuki Sumi

**Affiliations:** 1 Department of Clinical Information Applied Science, Institute of Science Tokyo, 1-5-45 Yushima, Bunkyo-ku, Tokyo 113-8519, Japan; 2 Department of Neurology and Neurological Science, Institute of Science Tokyo, 1-5-45 Yushima, Bunkyo-ku, Tokyo 113-8519, Japan; 3 Department of Laboratory Medicine, Institute of Science Tokyo, 1-5-45 Yushima, Bunkyo-ku, Tokyo 113-8519, Japan

**Keywords:** temporal deep learning, segmentation, peripheral nerve ultrasound, immune-mediated neuropathy, continuous ultrasound

## Abstract

**Objectives**: Peripheral nerve ultrasound is used to evaluate nerve enlargement in immune-mediated neuropathies; however, assessment can be challenging because the distribution and severity of nerve enlargement vary among patients and are often accompanied by indistinct nerve boundaries and heterogeneous echogenicity. Although deep learning-based segmentation has been reported, most studies have focused on limited regions or single anatomical sites, primarily in compressive neuropathies. This study aimed to evaluate the performance of temporal deep learning-based segmentation for assessing diffuse or focal nerve enlargement in immune-mediated neuropathies using continuous ultrasound scans. **Methods**: Twenty-five healthy participants and five patients with immune-mediated neuropathy and nerve enlargement were included. Continuous ultrasound scanning from the wrist to below the elbow was performed. A static DeepLabV3+ model and temporal models incorporating convolutional long short-term memory (ConvLSTM) or Temporal Mamba were constructed and compared. **Results**: In healthy participants, segmentation performance was comparable across models. In contrast, in patients with nerve enlargement, temporal models demonstrated higher Dice coefficients and reduced frame-to-frame variability. The ConvLSTM-based model showed the highest performance, with mean Dice coefficients ranging from 0.87 to 0.92. **Conclusions**: Temporal deep learning showed potential for nerve segmentation in selected cases with nerve enlargement associated with immune-mediated neuropathies. Temporal models achieved improved segmentation performance and reduced frame-to-frame variability in these preliminary cases. This approach may facilitate more consistent quantitative ultrasound evaluation and warrants further validation in larger cohorts.

## 1. Introduction

Peripheral nerve ultrasound is increasingly used as a complementary tool for the evaluation of peripheral neuropathies, particularly for detecting nerve enlargement in acquired demyelinating conditions such as chronic inflammatory demyelinating polyradiculoneuropathy (CIDP) [1,2,3,4]. In clinical practice, assessment of nerve enlargement relies on cross-sectional area (CSA) measurements; however, these are typically obtained from selected static frames and may be influenced by probe positioning and site selection. Although nerve ultrasound has relatively high intra-rater reliability, inter-rater variability remains a concern [5,6,7,8]. This issue becomes more pronounced in immune-mediated neuropathies, in which nerve enlargement is often diffuse or multifocal and accompanied by heterogeneous internal structure and variable echogenicity, making delineation of the epineurium difficult and increasing the likelihood of measurement variability [9,10]. Therefore, approaches that enable more objective and reproducible evaluation across the entire course of the nerve are clinically needed.

Recently, several studies have reported deep learning-based segmentation methods for nerve ultrasound images, achieving accuracies with Dice coefficients of approximately 0.8–0.9, suggesting the potential to improve measurement accuracy and reduce variability [11,12,13,14,15,16,17,18,19,20]. However, most of these studies have focused on limited regions or single anatomical sites, and many have been conducted in the context of carpal tunnel syndrome (CTS). Even in studies using video data, the ultrasound images are often obtained with the probe fixed at the wrist, capturing the median nerve moving and deforming during finger and wrist motion. In CTS, the pathology mainly involves nerve edema caused by compressive neuropathy, and nerve enlargement is typically focal and relatively uniform [21].

In contrast, in immune-mediated neuropathies, lesions may be distributed widely along peripheral nerves, and nerve ultrasound examinations are typically performed by moving the probe from the wrist toward the proximal arm. In these wide-range scanning images, the course of the nerve, its depth, and its positional relationship with surrounding tissues vary substantially, making the segmentation task different from that in previous studies. Furthermore, in continuous ultrasound scanning, adjacent frames are highly correlated, and ambiguous boundaries and speckle noise often persist across consecutive frames. Therefore, segmentation based on single-frame analysis may be insufficient, and incorporating temporal dependency is expected to improve robustness and stability.

To date, segmentation methods for scanning images of the median nerve from the wrist to below the elbow have been reported only in healthy participants and patients with CTS [22,23]. Segmentation studies for continuous forearm ultrasound scanning images in patients with diffuse or heterogeneous nerve enlargement remain limited. The aim of this study was to evaluate the feasibility and clinical utility of deep learning-based segmentation of continuous ultrasound scans of peripheral nerves from the wrist to below the elbow in immune-mediated neuropathies with heterogeneous enlargement and echogenicity patterns. We hypothesized that incorporating temporal dependency may improve segmentation performance and stability in continuous ultrasound scans, particularly in cases with heterogeneous nerve enlargement.

## 2. Materials and Methods

### 2.1. Study Participants and Nerve Ultrasound Protocol

Twenty-five healthy participants (17 men, 8 women; mean age, 37.2 ± 8.5 years) and 5 patients with immune-mediated neuropathies presenting with nerve enlargement participated in this study. The presence of nerve enlargement was determined based on the upper limit of normal CSA (mean + 2 standard deviations [SD]) calculated at each measurement site of the bilateral median and ulnar nerves in 25 healthy adults, with reference to a previous report [24]. The mean ± SD and upper limit of normal (mean + 2 SD) CSA values at each measurement site are shown in Supplementary Table S1. Age, sex, sites of nerve enlargement, and clinical diagnoses based on the EFNS/PNS 2021 diagnostic criteria [3] are shown in Table 1. This study was approved by the Ethics Committee of the Institute of Science Tokyo (#M2022-331). All participants provided written informed consent.

Ultrasound video sequences of the median and ulnar nerves from the wrist to below the elbow were acquired using an Aplio i800 ultrasound system (Canon Medical Systems, Tochigi, Japan) with an i18LX5 linear transducer. The frame rate was 60–65 fps and the imaging depth was set at 2 cm. The probe was moved continuously along the course of the nerve from the wrist to below the elbow. Nerve ultrasound examinations were performed by 2 neurologists.

### 2.2. Data Construction and Preprocessing

Ultrasound video sequences were acquired in DICOM format, and individual frames were exported as still images and converted into grayscale PNG images for analysis. The stored frame images were used for analysis and served as input data independently of the real-time display settings during acquisition. To correct tonal variations generated during device output, a constant intensity correction was applied to the stored images. The same preprocessing procedure was applied to all datasets used for training, validation, and evaluation. The input images were normalized to a range of 0–1 before being fed into the model.

From the acquired ultrasound video sequences, three board-certified neurologists with experience in neuromuscular ultrasound manually annotated the nerve regions at intervals of 10–15 frames. The annotation workload was divided among the neurologists. To ensure consistency across the entire dataset, all annotations were subsequently reviewed, and minor adjustments were made by the corresponding author (M.A.) when necessary. When both the median and ulnar nerves were visible in a single image, both nerves were annotated. The ultrasound images obtained from 25 healthy participants were divided into a training set (15 participants, 60 nerves, 4061 frames), a validation set (5 participants, 10 nerves, 1481 frames), and a test set (5 participants, 10 nerves, 1306 frames). Among the healthy participants, normal anatomical variants were included, such as bifid median nerves (3 nerves), persistent median arteries (3 nerves), and cases in which both the median and ulnar nerves were visualized in the same image (10 nerves). These variants were distributed across the training, validation, and test sets.

Because the number of patients was limited to 5, 3 patients (PT2, PT4, and PT5) were used for training (514 frames) and validation (240 frames), whereas 2 patients (PT1 and PT3) were reserved as an independent test set (394 frames). For the 3 patients used in training and validation, to avoid frame-level information leakage, consecutive images acquired on the same examination day were not divided across different dataset splits. Validation data consisted of images acquired on different days or from the contralateral nerves. Furthermore, to increase the influence of patient data in the training set, oversampling was applied by sampling the same patient data twice within the training dataset.

All images were resized to 480 × 288 pixels, and the model input consisted of single-channel grayscale images. Segmentation masks corresponding to each task were created as ground-truth labels.

### 2.3. Data Augmentation

To improve the generalization ability of the model, data augmentation was applied during training. Random affine transformations were used, including scaling (0.8–1.2), translation (±10%), rotation (±5°), and shear (±2°). To increase robustness to variations in image quality, contrast-limited adaptive histogram equalization (clip limit 1.0–2.0), gamma correction, and brightness and contrast adjustments were applied. In addition, Gaussian noise and Gaussian blur or motion blur (kernel size 3–7) were probabilistically applied to simulate noise and motion artifacts during ultrasound acquisition.

### 2.4. Model Architectures

#### 2.4.1. DeepLabV3+ (Image)

DeepLabV3+ was used as a static-image baseline model [25]. In this model, each frame was processed as an independent image, and temporal information was not utilized. The implementation of DeepLabV3+ was based on the segmentation–models–pytorch (smp) library [26]. The encoder was ResNet-34 pretrained on ImageNet, which is the default configuration in the smp library [27].

#### 2.4.2. DeepLabV3+ with Convolutional Long Short-Term Memory

**Figure 1:**
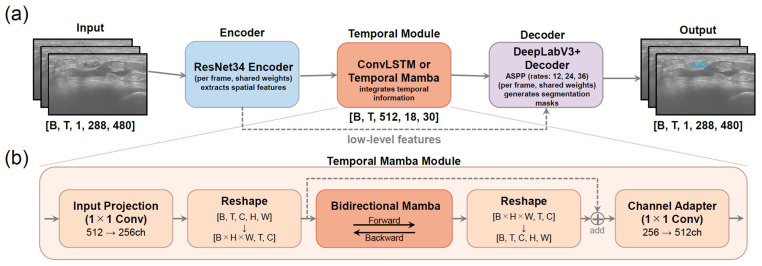
Architecture of the spatiotemporal integrated segmentation framework. (**a**) Overall architecture. An ultrasound video clip consisting of *T* frames is processed frame-by-frame by a weight-shared ResNet-34 encoder, which extracts spatial features and generates bottleneck feature maps [*B*, *T*, 512, 18, 30]. These features are then fed into a temporal module (convolutional long short-term memory or Temporal Mamba) to integrate temporal dependencies across frames. The temporally integrated features are subsequently input to the DeepLabV3+ decoder (ASPP), which aggregates multi-scale contextual information and outputs segmentation masks for each frame [*B*, *T*, 1, 288, 480]. (**b**) Detailed structure of the Temporal Mamba module. Channel compression is first applied to the encoder output using a 1 × 1 convolution, followed by tensor reshaping for temporal sequence processing. A bidirectional Mamba block integrates temporal information from both forward and backward directions. The features are then reshaped back into the spatial format. Finally, a channel adaptation layer is applied, and the output is combined with the input features through a residual connection. ConvLSTM follows the standard architecture described by Shi et al. [28] and is therefore not illustrated in detail.

In contrast to the static baseline model, a model incorporating temporal information was constructed based on DeepLabV3+, in which temporal modeling was introduced at the bottleneck level (Figure 1a). Specifically, features were extracted from each frame using a shared encoder, and only the deepest feature maps were temporally integrated using a bidirectional convolutional long short-term memory (ConvLSTM) [28], after which the outputs were fed into a shared decoder and segmentation head. The ConvLSTM module used convolutional gates with a kernel size of 3 × 3, and 1 bidirectional layer was applied. The outputs from the forward and backward directions were concatenated along the channel dimension and fused to the original number of channels using a 1 × 1 convolution. The number of hidden channels was set to 256, and no residual connections were used. The input consisted of clips composed of consecutive frames. Each clip contained 8 frames (frame size = 8). No frame overlap was introduced between adjacent clips (overlap size = 0). Each clip was input into the model while preserving the temporal order, and a ConvLSTM was applied to the features extracted by DeepLabV3+. The bidirectional ConvLSTM module contained approximately 14.3 million parameters and required approximately 123 GFLOPs per 8-frame clip.

#### 2.4.3. DeepLabV3+ with Temporal Mamba

Instead of ConvLSTM, a bidirectional Mamba module [29] based on a state-space model (SSM) was used to model temporal features. The bidirectional Mamba module was applied to the bottleneck features of DeepLabV3+, enabling efficient integration of long-range temporal dependencies across consecutive frames (Figure 1a,b).

The design was inspired by the bidirectional Mamba module in ViViM [30]. However, unlike ViViM, which jointly processes spatial and temporal information, spatial feature extraction was handled by the convolutional neural network backbone, and temporal modeling was applied independently at each spatial location of the bottleneck feature maps.

The bottleneck feature maps with dimensions [*B*, *T*, *C*, *H*, *W*] (*B*, batch size; *T*, number of frames; *C*, number of channels; *H* and *W*, spatial resolution) were reshaped into [*B* × *H* × *W*, *T*, *C*], allowing the sequence of *T* feature vectors at each spatial location to be used as the input sequence for the Mamba SSM. The state dimension (d_state) was set to 16, the local convolution width (d_conv) to 4, and the expansion factor (expand) to 2. One bidirectional layer was applied with residual connections. The input clip configuration and number of frames were identical to those used in the LSTM-based video model. The bidirectional Temporal Mamba module contained approximately 1.1 million parameters and required approximately 10 GFLOPs per 8-frame clip.

### 2.5. Training Settings

All experiments were conducted on a workstation equipped with an Intel Core Ultra 9 285K CPU (3.7 GHz, 64 GB RAM) and an NVIDIA GeForce RTX 5090 GPU (32 GB VRAM). All models were trained using the AdamW optimizer with parameters β_1_ = 0.9, β_2_ = 0.999, and weight_decay = 0.01. The DiceCELoss class from the MONAI framework [31] was used as the loss function. By enabling the jaccard = True option, the Dice loss component was replaced with Jaccard loss. The loss function was defined as a linear combination of Jaccard loss and cross-entropy loss. Because the nerve region is relatively small and class imbalance is substantial, the weights of Jaccard loss and cross-entropy loss were set to 0.8 and 0.2, respectively, to emphasize region overlap.

The learning rate was set to 1 × 10^−4^ for all models. The batch size was 8 for the static image model DeepLabV3+ (image). For the temporal models, DeepLabV3+ with ConvLSTM and DeepLabV3+ with Temporal Mamba, the batch size was set to 3 owing to GPU memory limitations. All models were implemented using PyTorch (version 2.9.1) [32]. PyTorch Lightning (version 2.5.6) was used for training management, and the MONAI framework (version 1.5.1) was used for the implementation of the loss functions. Only annotated frames were used for training and evaluation. Temporal clips were constructed exclusively from annotated frames, and intermediate unlabeled frames were not included in the training process. Losses were computed for all labeled frames within each temporal clip.

### 2.6. Evaluation Metrics

All models were trained for up to 100 epochs. During training, the Dice coefficient on the validation dataset was calculated at the end of each epoch, and the model weights corresponding to the highest validation Dice score were saved. The model selected based on the best validation Dice score was used for the final quantitative evaluation and visualization.

### 2.7. Additional Analyses

Additional analyses were conducted for the temporal models used in the primary analysis. Training was performed by adding boundary loss (weight = 0.01) [33] to DiceCELoss in order to evaluate the effect of a boundary-aware loss function on segmentation performance. Boundary loss was computed by generating a signed Euclidean distance map from the target mask and calculating its spatial inner product with the predicted probabilities. The distance map was calculated at the pixel level, and no correction based on physical resolution was applied.

Furthermore, to address the misclassification of vessels adjacent to the nerves, such as persistent median arteries, into nerve structures, multi-class segmentation with 3 classes (nerve, vessel, and background) was additionally investigated using a dataset with additional vessel annotations.

## 3. Results

### 3.1. Model Comparison

Table 2 shows the frame-averaged Dice coefficients for normal nerves and enlarged patient nerves. The frame-averaged values for each nerve in the 5 healthy participants are presented in Supplementary Table S2.

In normal nerves, the mean ± SD Dice coefficients of DeepLabV3+ (image), DeepLabV3+ with ConvLSTM, and DeepLabV3+ with Temporal Mamba were 0.877 ± 0.041, 0.884 ± 0.032, and 0.873 ± 0.049 for the median nerve and 0.859 ± 0.027, 0.872 ± 0.012, and 0.869 ± 0.017 for the ulnar nerve, respectively. All models showed good segmentation performance, and no clear differences in Dice coefficients were observed among the 3 models.

In contrast, higher Dice coefficients were observed in the temporal models for the enlarged nerves of PT1. For the right median nerve, the Dice coefficient improved from 0.816 ± 0.181 (image) to 0.914 ± 0.030 (ConvLSTM) and 0.894 ± 0.059 (Temporal Mamba). Similar tendencies toward higher Dice coefficients with temporal models were observed in the left median nerve and the bilateral ulnar nerves. DeepLabV3+ with ConvLSTM showed the highest mean Dice coefficient and the smallest standard deviation, indicating reduced variability in segmentation across frames.

Intersection over union (IOU), precision, and recall showed trends similar to those observed for the Dice coefficient, with higher values generally observed in the temporal models for enlarged nerves (Table 3, Supplementary Tables S3 and S4).

**Figure 2:**
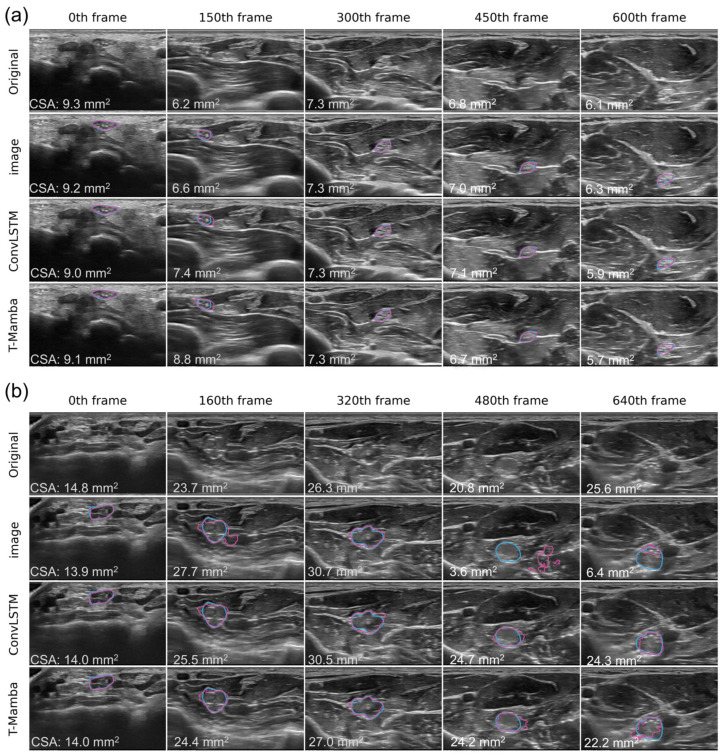
Segmentation results of the median nerve from the wrist to below the elbow. Cyan contours indicate the ground truth, and magenta contours indicate the predicted segmentation. Frame numbers are shown at the top of each column, with frame 0 corresponding to the wrist level; increasing frame numbers indicate movement in the proximal direction. Representative frames were selected at approximately equal intervals across the entire video sequence. The cross-sectional area (CSA) is shown in the lower left corner of each frame. In the original images, CSA derived from the ground truth is shown, whereas in the model outputs, CSA calculated from the predicted masks is shown. (**a**) Healthy participant (HP1, 35-year-old male). No clear differences were observed among the three models. (**b**) PT1, right median nerve. At frames 160, 480, and 640, temporal models achieved higher accuracy than the static model, with DeepLabV3+ with convolutional long short-term memory (ConvLSTM) showing the best performance. Image: DeepLabV3+; ConvLSTM: DeepLabV3+ with ConvLSTM; T-Mamba: DeepLabV3+ with Temporal Mamba.

**Figure 3:**
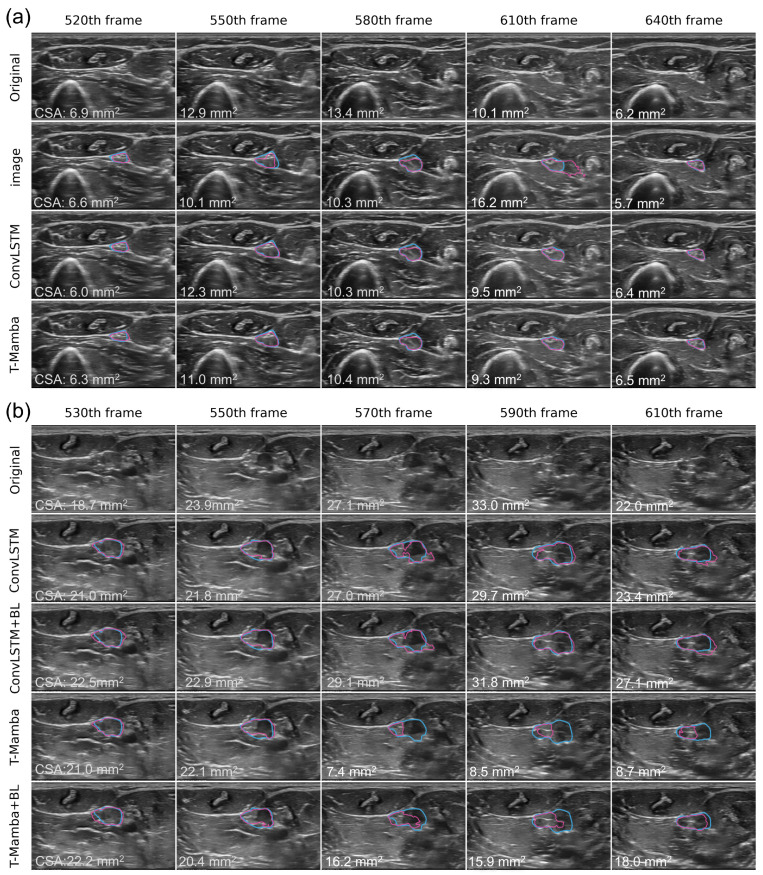
Segmentation results of the ulnar nerve. Cyan contours indicate the ground truth, and magenta contours indicate the predicted segmentation. Frame numbers are shown at the top of each column, with frame 0 corresponding to the wrist level; increasing frame numbers indicate movement in the proximal direction. The cross-sectional area (CSA) is shown in the lower left corner of each frame. In the original images, CSA derived from the ground truth is shown, whereas in the model outputs, CSA calculated from the predicted masks is shown. (**a**) PT3, right ulnar nerve at the forearm showing focal nerve enlargement. Localized enlargement is observed around frames 550, 580, and 610. The static model shows unstable contours and over-segmentation, whereas temporal models provide more stable contours across consecutive frames. (**b**) Effect of boundary loss on temporal models in the right ulnar nerve of PT1. At frames 570, 590, and 610, no substantial difference is observed in ConvLSTM with or without boundary loss. In Temporal Mamba, under-segmentation is observed, but boundary loss improves the segmentation. However, agreement with the ground truth remains lower than that of ConvLSTM. Image: DeepLabV3+; ConvLSTM: DeepLabV3+ with ConvLSTM; T-Mamba: DeepLabV3+ with Temporal Mamba. BL: boundary loss.

Representative segmentation results are shown in Figure 2a,b for a healthy participant (HP1, 35-year-old male) and the right median nerve of PT1 scanned from the wrist to below the elbow. In PT1, the difference between the static and temporal models was particularly apparent around frames 480 and 640. In these regions, the enlarged median nerve coursed deeper within the forearm. The nerve boundaries were already obscured by nerve enlargement and heterogeneous echogenicity, and the additional ultrasound attenuation associated with greater depth further reduced boundary visibility. Although the static model showed marked segmentation errors, the temporal models maintained more stable contour tracking. In addition, segmentation results of a focal enlargement of the right ulnar nerve in PT3 are presented in Figure 3a. In this localized nerve enlargement, the static image model DeepLabV3+ (image) showed unstable contours and over-segmentation, whereas the temporal models produced more stable contours across consecutive frames. Although no clear improvement in the overall Dice coefficient or IOU was observed in PT3, most scanned frames represented morphologically normal nerve segments because the enlargement was focal.

Overall, temporal models demonstrated improved segmentation performance and reduced variability compared with the static model, particularly in enlarged nerves.

### 3.2. Additional Analyses

#### 3.2.1. Temporal Models with Boundary Loss

**Figure 4:**
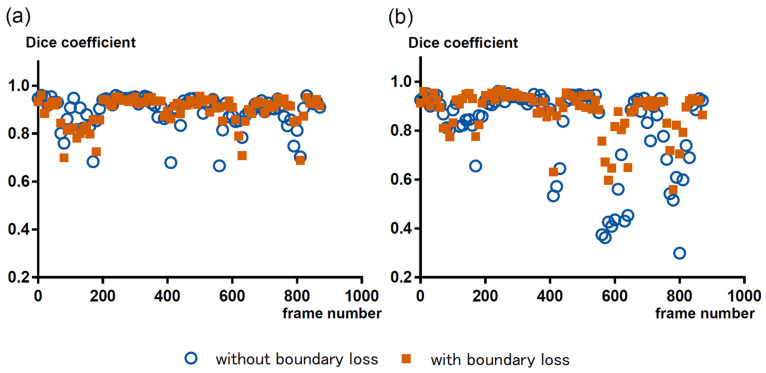
Frame-wise changes in Dice coefficients with boundary loss in the right ulnar nerve of PT1. The horizontal axis represents the frame number (distal to proximal), and the vertical axis represents the Dice coefficient. (**a**) DeepLabV3+ with convolutional long short-term memory (ConvLSTM). Boundary loss improved performance in some frames, but no consistent improvement was observed. (**b**) DeepLabV3+ with Temporal Mamba. Improvements were more apparent in frames with low Dice scores, suggesting a greater effect of boundary loss; however, overall performance did not exceed that of ConvLSTM.

Boundary loss (0.01) was added to the DeepLabV3+ with temporal models. Figure 4 shows the changes in Dice coefficients for each frame of the right ulnar nerve in PT1 with the addition of boundary loss in DeepLabV3+ with ConvLSTM and DeepLabV3+ with Temporal Mamba. In the ConvLSTM model, boundary loss improved the Dice coefficient in some frames, but without a clear, consistent improvement trend. In contrast, in the Temporal Mamba model, improvements in low-Dice frames were more evident, suggesting a relatively greater effect of boundary loss. However, the overall performance of Temporal Mamba did not surpass that of ConvLSTM. Segmentation results of the forearm portion of the right ulnar nerve in PT1, where large changes in Dice coefficients were observed, are shown in Figure 3b. In frames 570, 590, and 610, the nerve became markedly hypoechoic, resulting in less distinct nerve boundaries and increased segmentation difficulty. These image characteristics likely contributed to the decrease in Dice coefficients observed, particularly in the Temporal Mamba model, whereas the ConvLSTM model was relatively less affected.

#### 3.2.2. Normal Anatomical Variants and Multiclass Segmentation

**Figure 5:**
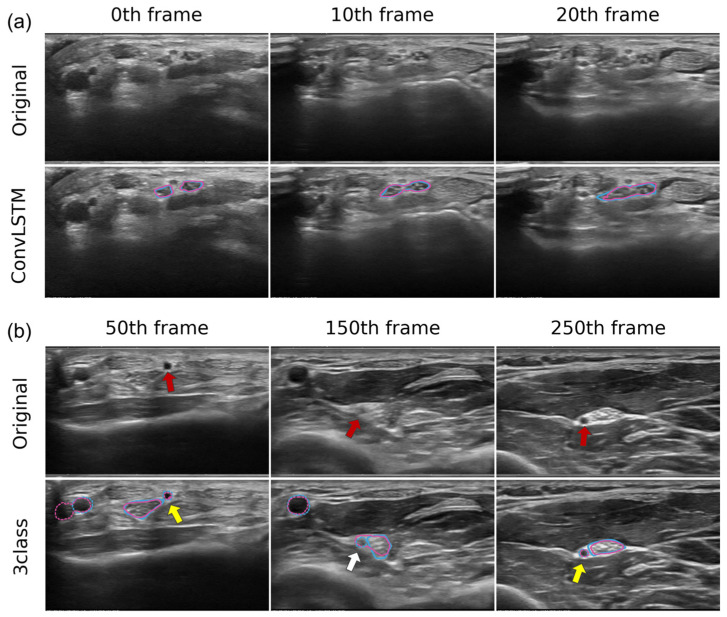
Segmentation results of normal anatomical variants. Cyan contours indicate the ground truth, and magenta contours indicate the predicted segmentation. Solid lines represent nerves, and dashed lines represent vessels. Frame numbers are shown at the top of each column, with frame 0 corresponding to the wrist level; increasing frame numbers indicate movement in the proximal direction. (**a**) Healthy participant (HP2), right median nerve. A bifid median nerve is observed at frames 0 and 10, with both branches segmented separately. (**b**) Healthy participant (HP3), right median nerve. Three-class segmentation results (DeepLabV3+ with ConvLSTM) in a case with a persistent median artery (red arrow). When the artery shows low echogenicity, it is correctly identified (yellow arrows, frames 50 and 250). When echogenicity is high and the boundary becomes indistinct, it is misclassified as a nerve (white arrows).

The bifid median nerve, a normal anatomical variant observed in a healthy participant (HP2), was also successfully identified by the model (Figure 5a).

Furthermore, to address the misclassification of vessels adjacent to the nerves as nerve structures, multiclass segmentation with 3 classes (nerve, vessel, and background) was performed. Vessels such as persistent median arteries (Figure 5b, red arrows) were correctly recognized as vessels when they showed lower echogenicity in comparison with the nerve (yellow arrows). However, when the echogenicity increased because of probe manipulation and became similar to that of the nerve, they were misclassified as nerves (white arrows) (Figure 5b, HP3).

## 4. Discussion

In ultrasound, peripheral nerves generally exhibit a honeycomb-like structure, and CSA is evaluated by tracing the hyperechoic epineurial boundary. In wide-range scanning such as that used in this study, the course, depth, and positional relationship of the nerve relative to surrounding structures change substantially. Therefore, nerve recognition requires not only local intensity information but also broader contextual information that includes surrounding anatomical structures. For this reason, in the present study, we adopted DeepLabV3+ [25] as the static image baseline model, as it can integrate contextual information while preserving spatial resolution through atrous convolution and atrous spatial pyramid pooling. Previous reports have also shown that DeepLabV3+ demonstrates better performance than U-Net and FPN in nerve ultrasound image segmentation [15]. In fact, in the present study, good results were obtained in healthy participant data using training based on static images alone.

However, in patients with nerve enlargement, identification of the nerve boundary becomes considerably more difficult owing to heterogeneous enlargement of nerve fascicles and variability of internal echogenicity, as well as increased muscle echogenicity caused by muscle degeneration. Under such conditions, single-frame estimation alone has limitations in handling ambiguous boundaries and morphological changes. In clinical practice, examiners identify nerves by referring to continuous structural changes observed during probe scanning. Based on this concept, we introduced models that incorporate temporal continuity. As a result, DeepLabV3+ with ConvLSTM and DeepLabV3+ with Temporal Mamba achieved mean Dice coefficients of approximately 0.85–0.92 even in cases with nerve enlargement. Compared with the static image model, frame-to-frame variability was reduced, suggesting improved stability of the segmentation. These findings suggest that automatic segmentation integrating temporal context may help address clinically important limitations in current nerve ultrasound evaluation, particularly in cases with diffuse and heterogeneous nerve enlargement.

In current clinical practice, CSA is typically measured only at representative sites on static images, and the results may be influenced by the selection of measurement sites. By applying automatic segmentation to continuous ultrasound scanning, CSA can be calculated continuously at multiple sites along the course of the nerve, which may reduce the influence of the subjective selection of measurement sites. Furthermore, this approach may enable the development of new quantitative indices, such as the distribution pattern of CSA, maximum CSA values, and the range of variation along the nerve.

ConvLSTM [28] is a method based on convolutional operations that captures local temporal dependencies between adjacent frames and is well suited for modeling short-term temporal continuity. In contrast, Mamba [29] is a sequence modeling method based on an SSM that sequentially integrates temporal information through input-dependent state transitions. Unlike Transformers [34], Mamba can model long-range dependencies while maintaining relatively low computational cost, and the utility of Mamba-based models has also been reported in ultrasound imaging in other organs [35]. The Temporal Mamba module used in this study differs from architectures such as the recently proposed U-Mamba [36], which incorporates SSMs in the spatial domain as well. Instead, spatial feature extraction was handled by a convolutional neural network backbone, while the SSM was applied specifically to temporal dependency modeling.

In this study, DeepLabV3+ with ConvLSTM showed better performance than DeepLabV3+ with Temporal Mamba. In the nerve ultrasound video sequences analyzed in this study, the frame rate was relatively high (60–65 fps), and probe movement during scanning was relatively slow; therefore, morphological changes between consecutive frames were relatively small. This finding further supports the notion that, in continuous ultrasound imaging with high frame rates and subtle inter-frame variations, modeling local temporal dependency is more effective than capturing long-range dependencies. On the other hand, indistinct boundaries and speckle noise tended to appear with similar patterns across multiple frames. Under these conditions, a ConvLSTM model that effectively utilizes local temporal consistency between adjacent frames may have been advantageous compared with models designed to capture long-range temporal dependencies. In addition, the relatively small dataset size may also have influenced model performance. In the additional analysis using boundary loss, improvements in low-Dice frames were more evident in the Temporal Mamba model, whereas no consistent improvement was observed with ConvLSTM. This may be because ConvLSTM already produced relatively stable contour estimation, limiting the additional effect of a boundary-aware loss function. In the future, when imaging conditions such as probe manipulation, scanning range, or ultrasound devices differ, models such as Temporal Mamba that can integrate longer-range temporal dependencies may show better performance. In addition, model capacity may also have influenced the observed differences in performance. The ConvLSTM model had substantially greater capacity compared with the Temporal Mamba model. Despite this difference, the two temporal models demonstrated broadly comparable segmentation performance on the present dataset. These findings suggest that the observed benefit of temporal modeling is unlikely to be explained solely by differences in model capacity.

In this study, normal anatomical variants, including bifid median nerve and persistent median artery, which have been excluded from analysis or reported as sources of oversight or misrecognition in previous studies [37], were also included in the training and evaluation. By training the model on images containing multiple nerves within a single frame, the bifid median nerve could also be recognized. Furthermore, because persistent median arteries have a fascicular structure similar to that of nerves, we also attempted a 3-class model in which vessels were explicitly learned. However, when the echogenicity of vessels was similar to that of nerves, misclassification still occurred, indicating that distinguishing small vessels adjacent to nerves remains a challenge.

This study has some limitations. First, the study was conducted at a single center, with a relatively consistent imaging protocol and relatively slow probe scanning speed. Therefore, further validation will be required to assess generalizability under different institutional settings, equipment conditions, or faster scanning conditions. In addition, CIDP is a relatively rare immune-mediated neuropathy, with a reported prevalence of approximately 3.3 per 100,000 and an incidence of approximately 0.36 per 100,000 person-years based on a recent nationwide study in Japan [38]. Autoimmune nodopathies represent an even smaller subset of CIDP [3]. Diffuse or multifocal nerve enlargement detectable on ultrasound is not observed in all of these patients. Therefore, the number of patients with nerve enlargement in this study was limited (n = 5). Despite the small patient cohort, our spatiotemporal analysis of thousands of continuous ultrasound frames—encompassing both healthy participants and patients—provides initial evidence supporting the feasibility. It suggests that temporal modeling may improve segmentation performance in some challenging pathological cases while maintaining stable performance across both normal nerves and selected cases with nerve enlargement. Moreover, because the data consisted of temporally continuous ultrasound frames with intra-sequence correlations, statistical comparisons assuming independent observations were not performed. Nevertheless, the results of this study suggest that automatic segmentation using deep learning has potential for practical clinical application, even in patients with nerve enlargement where boundaries are indistinct and morphological variability is large. With the accumulation of more diverse clinical images in the future, this approach is expected to contribute to quantitative evaluation and improved reproducibility in nerve ultrasound examinations.

From a clinical perspective, the ability to perform stable segmentation across continuous ultrasound scans may enable more objective and comprehensive evaluation of nerve morphology along its entire course. This approach may reduce operator dependency in site selection and facilitate the development of novel quantitative indices, such as spatial distribution patterns of nerve enlargement and variability along the nerve. In addition, it may provide a foundation for future real-time or longitudinal ultrasound analysis.

## 5. Conclusions

Temporal deep learning showed potential for nerve segmentation in selected cases of immune-mediated neuropathies with nerve enlargement. In preliminary patient cases, temporal models achieved higher segmentation performance and reduced frame-to-frame variability compared with the static image model. This approach may facilitate more consistent quantitative ultrasound evaluation in clinical practice. These findings support the feasibility of incorporating temporal modeling into the analysis of continuous ultrasound data.

## Figures and Tables

**Table 1:** Clinical diagnosis and distribution of nerve enlargement in patients with immune-mediated neuropathy.

ID	Age/Sex	Clinical Diagnosis	Distribution of Nerve Enlargement
PT1	20 M	Nodopathy (anti-NF155 antibody positive)	Diffuse (proximal dominant)
PT2	40 F	Typical CIDP	Diffuse (proximal dominant)
PT3	20 F	Multifocal CIDP	Rt ulnar at middle of forearm and below elbow
PT4	30 F	Possible multifocal CIDP	Lt ulnar below and above elbow
PT5	60 M	Possible focal CIDP	Rt ulnar at middle of forearm

CIDP, chronic inflammatory demyelinating polyradiculoneuropathy.

**Table 2:** Frame-averaged Dice coefficients for normal nerves and enlarged patient nerves.

Participant		Nerve	DeepLabV3+ (Image)	DeepLabV3+ with ConvLSTM	DeepLabV3+ with Temporal Mamba
Healthy participants (*n* = 5; 10 nerves)		Median	0.877 ± 0.041	0.884 ± 0.032	0.873 ± 0.046
		Ulnar	0.859 ± 0.027	0.872 ± 0.012	0.869 ± 0.017
PT1	Right	Median	0.816 ± 0.181	0.914 ± 0.030	0.894 ± 0.059
		Ulnar	0.813 ± 0.181	0.893 ± 0.066	0.815 ± 0.173
	Left	Median	0.812 ± 0.221	0.917 ± 0.042	0.895 ± 0.069
		Ulnar	0.785 ± 0.215	0.871 ± 0.090	0.841 ± 0.114
PT3	Right	Ulnar	0.872 ± 0.076	0.879 ± 0.064	0.866 ± 0.091

Data are presented as mean ± SD. ConvLSTM, convolutional long short-term memory.

**Table 3:** Frame-averaged intersection over union for normal nerves and enlarged patient nerves.

Participant		Nerve	DeepLabV3+ (Image)	DeepLabV3+ with ConvLSTM	DeepLabV3+ with Temporal Mamba
Healthy participants (*n* = 5; 10 nerves)		Median	0.792 ± 0.056	0.797 ± 0.049	0.786 ± 0.060
		Ulnar	0.762 ± 0.032	0.779 ± 0.017	0.770 ± 0.025
PT1	Right	Median	0.721 ± 0.208	0.843 ± 0.050	0.813 ± 0.088
		Ulnar	0.718 ± 0.214	0.813 ± 0.099	0.718 ± 0.210
	Left	Median	0.725 ± 0.232	0.849 ± 0.069	0.816 ± 0.103
		Ulnar	0.686 ± 0.233	0.781 ± 0.124	0.740 ± 0.152
PT3	Right	Ulnar	0.781 ± 0.105	0.789 ± 0.095	0.773 ± 0.123

Data are presented as mean ± SD. ConvLSTM, convolutional long short-term memory.

## Data Availability

The datasets generated and analyzed during the current study are not publicly available due to ethical and institutional restrictions but are available from the corresponding author upon reasonable request.

## Supplementary Materials

The following supporting information can be downloaded at https://www.mdpi.com/article/10.3390/diagnostics16121934/s1, Table S1: Normal cross-sectional area (CSA) values of the median and ulnar nerves at each measurement site; Table S2: Frame-averaged Dice coefficients for normal nerves and enlarged nerves among healthy participants. Table S3: Frame-averaged precision for normal nerves and enlarged nerves of patients. Table S4: Frame-averaged recall for normal nerves and enlarged nerves of patients.
