# Supplementary material for: Automated Segmentation of Diffuse and Multifocal Nerve Enlargement in Immune-Mediated Neuropathy Using Temporal Deep Learning on Continuous Ultrasound Scans
Source: Diagnostics (Basel). 2026 Jun 22;16(12):1934. doi: 10.3390/diagnostics16121934 (PMC13298017; doi:10.3390/diagnostics16121934)
Supplement: Supplementary file 1 [file diagnostics-16-01934-s001.zip › diagnostics-4331290-supplementary.pdf]

Table S1. Normal cross-sectional area (CSA) values of the median and ulnar nerves at each measurement site.

Nerve	Site	Mean (mm^2)	SD	Upper limit (mean + 2 SD)
Median	Wrist	8.3	1.9	12.1
	Forearm	6.0	1.2	8.3
	At the elbow	9.4	2.2	14.0
	Upper arm	8.7	1.6	11.9
Ulnar	Wrist	5.0	1.3	7.6
	Forearm	5.1	1.1	7.4
	Below the elbow	5.5	1.1	7.7
	At the elbow	6.6	2.4	11.4
	Above the elbow	5.3	1.4	8.0
	Upper arm	5.5	1.2	7.9

Median nerve: wrist, distal wrist crease; forearm, midpoint between the distal wrist crease and the antecubital fossa; elbow, antecubital fossa; upper arm, 10 cm proximal to the antecubital fossa. Ulnar nerve: wrist, distal wrist crease; forearm, midpoint between the distal wrist crease and the ulnar groove; below the elbow, 4 cm distal to the ulnar groove; at the elbow; ulnar groove; above the elbow, 6 cm proximal to the ulnar groove; upper arm, 16 cm proximal to the ulnar groove. SD, standard deviation.

Table S2. Frame-averaged Dice coefficients for normal nerves and enlarged nerves among healthy participants.

Participant	Side	Nerve	DeepLabV3+ (image)	DeepLabV3+ with ConvLSTM	DeepLabV3+ with Temporal Mamba
HP1	Right	Median	0.929 ± 0.024	0.925 ± 0.028	0.919 ± 0.037
		Ulnar	0.883 ± 0.093	0.887 ± 0.065	0.872 ± 0.101
	Left	Median	0.932 ± 0.026	0.930 ± 0.034	0.931 ± 0.029
		Ulnar	0.861 ± 0.099	0.870 ± 0.071	0.867 ± 0.067
HP2	Right	Median	0.870 ± 0.060	0.849 ± 0.081	0.847 ± 0.077
		Ulnar	0.848 ± 0.131	0.878 ± 0.050	0.883 ± 0.051
	Left	Median	0.899 ± 0.038	0.899 ± 0.040	0.892 ± 0.054
		Ulnar	0.867 ± 0.058	0.875 ± 0.073	0.889 ± 0.041
HP3	Right	Median	0.850 ± 0.088	0.850 ± 0.079	0.773 ± 0.247
		Ulnar	0.869 ± 0.052	0.871 ± 0.046	0.854 ± 0.050
	Left	Median	0.898 ± 0.042	0.896 ± 0.049	0.903 ± 0.044
		Ulnar	0.852 ± 0.058	0.865 ± 0.053	0.844 ± 0.070
HP4	Right	Median	0.866 ± 0.062	0.865 ± 0.060	0.865 ± 0.049
		Ulnar	0.871 ± 0.055	0.871 ± 0.052	0.868 ± 0.059
	Left	Median	0.865 ± 0.068	0.876 ± 0.066	0.859 ± 0.076
		Ulnar	0.876 ± 0.037	0.885 ± 0.040	0.879 ± 0.038
HP5	Right	Median	0.788 ± 0.211	0.846 ± 0.073	0.842 ± 0.074
		Ulnar	0.789 ± 0.189	0.845 ± 0.083	0.834 ± 0.120
	Left	Median	0.878 ± 0.138	0.907 ± 0.040	0.902 ± 0.047
		Ulnar	0.871 ± 0.060	0.878 ± 0.061	0.868 ± 0.128

Data are presented as mean ± SD. ConvLSTM, convolutional long short-term memory.

Table S3. Frame-averaged precision for normal nerves and enlarged nerves of patients.

Participant		Nerve	DeepLabV3+ (image)	DeepLabV3+ with ConvLSTM	DeepLabV3+ with Temporal Mamba
Healthy participants (n = 5; 10 nerves)		Median	0.848 ± 0.053	0.858 ± 0.054	0.844 ± 0.056
		Ulnar	0.810 ± 0.053	0.856 ± 0.042	0.866 ± 0.042
PT1	Right	Median	0.849 ± 0.138	0.906 ± 0.058	0.900 ± 0.060
		Ulnar	0.910 ± 0.099	0.904 ± 0.085	0.916 ± 0.079
	Left	Median	0.853 ± 0.212	0.917 ± 0.085	0.917 ± 0.082
		Ulnar	0.882 ± 0.187	0.889 ± 0.088	0.905 ± 0.086
PT3	Right	Ulnar	0.839 ± 0.121	0.843 ± 0.111	0.852 ± 0.127

Data are presented as mean ± SD. ConvLSTM, convolutional long short-term memory.

Table S4. Frame-averaged recall for normal nerves and enlarged nerves of patients.

Participant		Nerve	DeepLabV3+ (image)	DeepLabV3+ with ConvLSTM	DeepLabV3+ with Temporal Mamba
Healthy participants (n = 5; 10 nerves)		Median	0.921 ± 0.045	0.925 ± 0.037	0.921 ± 0.060
		Ulnar	0.896 ± 0.045	0.904 ± 0.040	0.883 ± 0.057
PT1	Right	Median	0.832 ± 0.229	0.928 ± 0.053	0.897 ± 0.094
		Ulnar	0.779 ± 0.234	0.894 ± 0.096	0.781 ± 0.235
	Left	Median	0.796 ± 0.245	0.924 ± 0.050	0.886 ± 0.098
		Ulnar	0.735 ± 0.243	0.869 ± 0.132	0.800 ± 0.155
PT3	Right	Ulnar	0.926 ± 0.075	0.935 ± 0.074	0.903 ± 0.102

Data are presented as mean ± SD. ConvLSTM, convolutional long short-term memory.
